# Neighbourhood disadvantage, geographic remoteness and body mass index among immigrants to Australia: A national cohort study 2006-2014

**DOI:** 10.1371/journal.pone.0191729

**Published:** 2018-01-23

**Authors:** Karen Menigoz, Andrea Nathan, Kristiann C. Heesch, Gavin Turrell

**Affiliations:** 1 School of Public Health and Social Work and Institute of Health and Biomedical Innovation, Queensland University of Technology (QUT), Brisbane, Queensland, Australia; 2 Institute for Health & Ageing, Australian Catholic University (ACU), Melbourne, Victoria, Australia; National Institute of Health, ITALY

## Abstract

Obesity is socioeconomically, geographically and ethnically patterned. Understanding these elements of disadvantage is vital in understanding population obesity trends and the development of effective and equitable interventions. This study examined the relationship between neighbourhood socioeconomic disadvantage and geographic remoteness with prospective trends in mean body mass index (BMI) among immigrants to Australia. Longitudinal data (2006–2014) from a national panel survey of Australian adults was divided into an immigrant-only sample (n = 4,293, 52.6% women and 19,404 person-year observations). The data were analysed using multi-level random effects linear regression modelling that controlled for individual socioeconomic and demographic factors. Male immigrants living in the most disadvantaged neighbourhoods had significantly higher mean BMI compared with those living in the least disadvantaged. Over time, mean BMI increased for all groups except for men living in the least disadvantaged neighbourhoods, for whom mean BMI remained almost static (0.1 kg/m^2^ increase from 2006 to 2014), effectively widening neighbourhood inequalities. Among women, mean BMI was also significantly higher in the most compared with the least, disadvantaged neighbourhoods (β = 2.08 kg/m^2^; 95%CI: 1.48, 2.68). Neighbourhood inequalities were maintained over time as mean BMI increased for all groups at a similar rate. Male and female immigrants residing in outer regional areas had significantly higher mean BMI compared with those living in major cities; however, differences were attenuated and no longer significant following adjustment for ethnicity, individual socioeconomic position and neighbourhood disadvantage. Over time, mean BMI increased in all male and female groups with no differences based on geographic remoteness. Obesity prevention policy targeted at immigrant cohorts needs to include area-level interventions that address inequalities in BMI arising from neighbourhood disadvantage, and be inclusive of immigrants living outside Australia’s major cities.

## Introduction

Worldwide, adult overweight and obesity rose by 27.5% between 1980 and 2013, with an estimated 2.1 billion people overweight or obese in 2013 [1]. In parallel, global movements of people increased by 41% from 2000 to 2015, with 244 million people living outside their country of birth in 2015 [2]. Reviews and longitudinal studies have demonstrated persistent inequalities in the prevalence of overweight and obesity for ethnic groups in the United States (US) [3–5], Canada [6] and the United Kingdom [7]. It is therefore critical to develop an understanding of the drivers of obesity across all population groups to underpin efforts to halt the obesity epidemic.

Previous explorations of the reasons for ethnic inequalities in obesity have taken an individual-level approach: they have focused on individual risk factors, adaptation or acculturation processes, individual socioeconomic position, and cultural factors [8–12]. Alongside these compositional effects, researchers and theorists have asserted the need to consider broader contextual or area-level effects [5,13–16]. Contextual factors associated with obesity and body mass index (BMI) in ethnic minority groups include attributes of the built environment [16–20] and of the social environment [5,19,20], ethnic density [21–25], racial or residential segregation [26–28], neighbourhood socioeconomic disadvantage [28–32] and geographic remoteness (urban vs rural) [33]. The majority of these studies have been from the US, and wider research examining the role of contextual factors on immigrant obesity trends in other developed countries is needed to advance the field and guide policy development [12,21].

Neighbourhood socioeconomic disadvantage and geographic remoteness are particularly relevant to the study of overweight and obesity in Australia. In Australian studies with the general population, cross-sectional associations between neighbourhood socioeconomic disadvantage and higher BMI have been demonstrated among men and women living in more deprived areas compared with those living in less deprived areas [34,35]. The two known longitudinal studies [36,37] confirm and extend these findings demonstrating that neighbourhood inequalities in BMI are maintained over time (BMI increasing at a similar rate across all groups)[36] and that neighbourhood inequalities are maintained through age groups in men and widen with age in women [37]. Geographic remoteness is also important, because immigrants living in rural Australia may have poorer general well-being compared with immigrants living in urban areas [38]. Further, higher obesity prevalence has been documented in the general Australian population living in rural versus urban areas [39,40] and accumulated exposure to rural areas has been shown to result in higher obesity later in life [41].

Despite calls for population sub-group research to understand the role of neighbourhood context in vulnerable groups [5] and inform equitable health policy [42], no known studies have examined the double disadvantage which may arise for immigrants living in socioeconomically deprived neighbourhoods or geographically remote areas in Australia. This is a significant evidence gap for policy makers given that in 2016, 28.2% of the Australian population were born overseas [43]; we have demonstrated ethnic differences in overweight and obesity comparing overseas-born with native-born Australians [44]; choice of neighbourhood of residence is ethnically patterned [26]; and Australian immigration policy is promoting settlement in regional areas [45]. Further, obesity is a public health priority in Australia, as in 2014–15, 70.8% of men and 56.3% of women aged 18 years and over were overweight or obese [46], placing Australia in 5^th^ place for adult obesity prevalence in Organization for Economic Cooperation and Development (OECD) countries (behind the US, Mexico, New Zealand and Hungary) [47]. The current study contributes to addressing the identified gaps in the literature by using contemporary cohort data to examine BMI trends by neighbourhood disadvantage and geographic remoteness among immigrant men and women in Australia.

## Materials and methods

### Ethics statement

Permission was sought and granted to use the Household Income and Labour Dynamics in Australia (HILDA) *In Confidence Release* dataset for the purposes of this research, through an organisational licensing agreement between Queensland University of Technology and the Australian Government’s Department of Social Services, and through signing a Deed of Confidentiality. The HILDA survey has ethics approval from The Faculty of Business and Economics Human Ethics Advisory Committee (University of Melbourne) (reference number 1135382.4). Access to the HILDA *In Confidence Release* dataset was necessary in order to obtain the level of geographic detail required to examine neighbourhood effects. The level of geographic detail in the HILDA *In Confidence Release* had the potential to allow participants to be identified. As such, a negligible/low risk ethics approval was required. The research was reviewed and confirmed as meeting the requirements of the National Statement on Ethical Conduct in Human Research and has received ethics approval from the Human Research Ethics Committee at the Queensland University of Technology (reference number 1500000836).

### Study design and sample

This study was conducted using data from the HILDA survey, a national panel survey that began in 2001. The survey is administered annually by trained interviewers to all household members aged 15 years and over, who then self-complete a questionnaire. The HILDA reference population is all members of private dwellings in Australia. Details about study methods are published elsewhere [48]. Briefly, a multi-staged sampling methodology was used to select households who would form the survey panel. From a sample of 488 Census Collection Districts (CCD’s) across Australia (each CCD consists of approximately 200 to 250 households), a sample of 22 to 34 dwellings was selected and within each dwelling, up to three households were chosen. In the first wave this resulted in a probability sample of 7,682 households (19,914 individuals) and over time, the sample expands to include new members of survey households. In 2011, the sample was replenished, adding a further 2,153 households (5,477 individuals) using a similar recruitment methodology as the first wave [49]. Inclusion of the 2011 top-up sample has been shown to improve the representativeness of the data, particularly in relation to immigrants’ country of birth and length of residence in Australia [50]. The combined original and top-up samples have also improved the comparability of estimates against the Australian Bureau of Statistics Labour Force Survey [50]. Attrition analyses showed that study drop-out has been more likely for those who identify as Indigenous Australians, are single, unemployed or in low skilled occupations, were born in a non-English speaking country, or are young (aged 15–24 years) [51].

The current analysis used nine waves of data, beginning in 2006 (wave 6) when height and weight data were first collected, through to 2014 (wave 14). Observations from individuals aged less than 18 years (n = 8,880) and women (at any wave) who were pregnant in the previous year (n = 162) were excluded. As immigrants were the population group of interest for this study, people born in Australia were also excluded (n = 17,715), as were people who moved during the study period (n = 291), to allow for a consistent environmental exposure to neighbourhood disadvantage and geographic remoteness over time.

### Variables

BMI was calculated from height and weight (weight in kilograms divided by height in metres squared) reported by participants in the self-completed questionnaire. BMI was modelled as a continuous variable in all analyses so that interpretation of the data was not influenced by debate about different ethnic BMI cut-off points for overweight and obesity [52].

Neighbourhood disadvantage was operationalised as the SEIFA (Socio-Economic Indexes for Area) 2011 Index of Relative Socio-economic Disadvantage (IRSD) decile score [53]. IRSD scores are assigned by the Australian Bureau of Statistics to an area that contains an average population of approximately 400 persons (the geographical unit called Statistical Area Level 1)[54]. The IRSD is derived from 16 variables, including, the proportion of people who have low household income, are unemployed, have low status occupations, have no or low education levels, live in overcrowded or lower quality housing, are separated/divorced, have a disability or a long-term health condition, or do not speak English well; the proportion of families containing children living with jobless parents and one parent families with dependent offspring; and the proportion of residences with no internet connection and no cars [53]. For the analyses, the IRSD deciles were collapsed into quintiles of neighbourhood disadvantage with Quintile 1 denoting the most disadvantaged neighbourhoods, and Quintile 5 the least disadvantaged.

Geographic remoteness was assessed using the Australian Bureau of Statistics’ 2011 Australian Statistical Geography Standard (ASGS) Remoteness Structure [55]. The Remoteness Structure is also assigned at Statistical Area Level 1 and is a commonly used measure to divide Australia into areas that share common remoteness characteristics based on the road distance to services. As a measure of geographic remoteness and accessibility specific to the Australian context, it does not necessarily reflect an area’s rurality, socioeconomic characteristics nor population size [56]. For this study, four categories were used: major city, inner regional, outer regional and a combined category of remote and very remote (due to small sample sizes in each category).

Individual socioeconomic and demographic variables, collected from the survey questionnaires, were also used in the analysis. Baseline age was calculated as age in 2006 (mean-centred) and an age-squared variable, which was included due to our observation of a curvilinear association between age and BMI. Ethnicity was included in the analysis given that neighbourhood settlement patterns vary by ethnic group [26] and ethnicity is associated with BMI [44]. Ethnicity was assessed with the question, “In which country were you born?”, and responses were aggregated according to the Standard Australian Classification of Countries (a standard classification used by the Australian Bureau of Statistics that is based on similarities between countries in political, economic and social characteristics and also their geographic proximity) [57]. Individual-level socioeconomic indicators (household income, education and occupation) were included in the analyses in order to simultaneously model both individual-level socioeconomic factors and their area-level analogues [58]. Annual household disposable income was reported as total regular household income from all sources minus income tax. The highest attained education level was assessed from a series of interview questions. Occupation was coded according to the 4-digit Australian and New Zealand Standard Classification of Occupations (ANZSCO 2006) [59], based on responses to questions about participants’ occupation title and the tasks/duties undertaken in their job.

### Statistical analyses

The analytic sample was formed by excluding individuals who had implausible or incomplete BMI data (n = 480) or who were missing data on socio-economic characteristics (n = 4). For those missing BMI data, over 75% were excluded for non-return of the self-completed questionnaire, and non-return of the questionnaire has been shown to be more likely among those born overseas in countries where English is not the main language and those with low education levels [60]. The final analytic sample contained 4,293 individuals (52.4% women) and 19,404 person-year observations (52.6% women). An unbalanced panel, allowing respondents to leave and re-join the survey over the study period, was used in all analyses. Consistent with previous studies of obesity and area-level disadvantage in Australia [35–37], men and women were analysed separately in recognition of the gendered determinants of BMI. All analyses were conducted using STATA/SE Release 13 (College Station, TX: StataCorp LP) and MLwiN v2.27 [61].

Summary statistics (mean and standard deviation) on all variables at the first and final waves were computed. Longitudinal random effects modelling was used to assess prospective trends in mean BMI by neighbourhood disadvantage and geographic remoteness. Given that the survey sampling unit was the household, multilevel models were used to account for the hierarchical nature of the data structure with observations (waves) nested within individuals, who were nested within households, which were nested within CCD’s. A four-step modelling process was used to assess the impact of controlling for various factors on the coefficient estimates. Step 1 (model 1): BMI (the dependent variable) was regressed on neighbourhood disadvantage (the independent variable), with adjustment for age, age squared and survey year. Step 2 (model 2): the regression analysis from step 1 was repeated with the addition of the ethnicity variable to the model. Step 3 (model 3): the regression analysis from step 2 was repeated with the addition of geographic remoteness, education, occupation and household income. Model 3 coefficient estimates were used to assess the association between neighbourhood disadvantage and BMI after full adjustment. Step 4 (model 4): the regression analysis from step 3 was repeated with the addition of an interaction term of neighbourhood disadvantage by survey year, to assess whether the rate of change in BMI varied by neighbourhood disadvantage. In all models, Quintile 5 (the least disadvantaged neighbourhood) was the reference category, as this allowed for easier interpretation of the results as positive coefficients and a discussion of inequalities comparing the most disadvantaged neighbourhoods to the least disadvantaged.

A similar, four-step modelling process was undertaken with geographic remoteness as the independent variable. The only difference to the previous modelling was that in Step 3 (model 3), neighbourhood disadvantage was added as one of the control variables (in lieu of geographic remoteness). In all models, the reference category was major cities, as this allowed comparison with the theoretically least disadvantaged group.

## Results

The characteristics of the analytic sample in 2006 and 2014 (the first and final time points) are shown in Table 1. The mean BMI of men and women in 2014 was 27.2 kg/m^2^ (SD 4.8) and 25.8 kg/m^2^ (SD 5.6) respectively. Consistent with Australia’s immigrant profile, the predominant ethnicity of respondents was North-West European and Southern and Eastern European, followed by Oceania for men and South-East Asian for women. For men, there was a slightly higher proportion of the sample in the least disadvantaged neighbourhood (Quintile 5), and in 2014, men in Quintile 3 had the highest BMI (28.0 kg/m^2^ (SD 5.4)). Women in the sample were evenly distributed across quintiles of neighbourhood disadvantage and women in the most disadvantaged neighbourhood (Quintile 1) had the highest BMI in 2014 (27.2 kg/m^2^ (SD 6.0)). Approximately 80% of respondents lived in major cities, and BMI was highest in outer regional Australia for both men and women, exceeding 28 kg/m^2^ for both genders in 2014.

**Table 1:** Neighbourhood disadvantage, geographic remoteness, socio-demographic characteristics and mean body mass index of men and women in the analytic sample, 2006 and 2014.

* *	*Men (2006)*		*Men (2014)*		*Women (2006)*		*Women (2014)*	
* *	*(n = 891)*		*(n = 972)*		*(n = 1205)*		*(n = 1368)*	
* *	*%*	*Mean BMI (SD)*	*%*	*Mean BMI (SD)*	*%*	*Mean BMI (SD)*	*%*	*Mean BMI (SD)*
Overall		26.7 (4.4)		27.2 (4.8)		25.7 (5.4)		25.8 (5.6)
*Country of birth*^*a*^								* *
Oceania (excluding Australia)	11.5	27.9 (4.9)	15.2	29.1 (5.7)	9.9	26.6 (6.0)	11.3	27.3 (6.3)
North-West Europe	48.0	26.7 (4.1)	40.8	27.1 (4.3)	42.3	26.1 (5.1)	36.3	26.5 (5.8)
Southern & Eastern Europe	13.4	27.2 (4.0)	10.3	28.3 (4.3)	13.5	26.9 (5.1)	9.9	27.2 (6.2)
North Africa & Middle East	3.1	29.3 (8.3)	3.8	29.3 (7.7)	2.7	28.6 (7.8)	3.5	27.2 (6.2)
South-East Asia	6.9	25.3 (3.6)	8.7	25.4 (4.4)	12.1	24.0 (5.3)	13.9	24.1 (4.9)
North-East Asia	3.6	23.6 (3.2)	4.7	24.7 (4.3)	5.9	21.6 (3.0)	7.0	21.9 (2.8)
Southern & Central Asia	5.3	25.4 (3.4)	8.1	24.9 (3.4)	4.9	25.0 (4.1)	6.8	24.9 (4.1)
Americas	3.7	28.0 (5.7)	3.4	28.3 (4.3)	4.5	24.5 (4.2)	6.6	25.0 (4.5)
Sub-Saharan Africa	4.6	25.4 (3.5)	5.1	26.5 (4.1)	4.2	26.1 (6.5)	4.6	24.6 (4.9)
* Neighbourhood disadvantage*								
Quintile 5 (least disadv.)	25.6	26.3 (4.1)	24.6	26.6 (3.8)	21.8	24.2 (4.3)	23.1	24.2 (4.5)
Quintile 4	19.4	26.1. (3.6)	21.7	26.9 (4.4)	20.6	25.2 (5.1)	20.9	25.5 (5.0)
Quintile 3	18.0	27.2 (4.7)	16.4	28.0 (5.4)	18.9	26.0 (5.1)	17.5	25.7 (5.9)
Quintile 2	18.7	27.3 (5.5)	17.8	27.6 (5.7)	18.6	26.2 (5.9)	17.4	26.4 (6.0)
Quintile 1 (most disadv.)	18.3	27.0 (4.2)	19.6	27.3 (5.0)	20.1	27.2 (6.0)	21.1	27.2 (6.0)
*Remoteness*								
Major city	78.4	26.6 (4.4)	79.6	27.1 (4.8)	78.4	25.7 (5.5)	80.8	25.5 (5.4)
Inner regional	14.2	26.9 (4.7)	13.5	27.3 (4.3)	14.5	25.1 (4.4)	12.1	26.2 (5.8)
Outer regional	6.5	27.1 (4.1)	6.0	28.5 (5.7)	6.2	27.3 (6.1)	6.3	28.1 (6.9)
Remote and very remote	0.8	26.6 (2.8)	0.9	26.8 (4.0)	0.9	24.3 (2.6)	1.1	25.1 (6.6)
*Age*								
18–24 years	4.5	23.5 (3.8)	3.3	24.7 (5.2)	4.6	23.0 (5.8)	3.7	23.5 (5.5)
25–34 years	9.6	26.5 (4.3)	10.5	26.6 (4.9)	9.1	23.3 (4.0)	10.5	23.4 (4.7)
35–44 years	17.4	26.6 (5.3)	13.8	27.0 (4.7)	20.4	24.8 (5.8)	15.1	25.0 (5.4)
45–54 years	22.2	26.5 (3.9)	20.8	27.5 (5.4)	24.4	26.0 (5.5)	21.7	26.0 (5.8)
55–64 years	21.7	27.6 (4.5)	20.5	27.5 (4.5)	19.7	27.1 (5.1)	20.8	26.4 (5.5)
65–74 years	15.8	27.2 (3.9)	19.8	27.7 (4.6)	13.4	26.3 (4.8)	17.0	27.3 (5.7)
≥ 75 years	8.8	26.1 (4.3)	11.4	27.0 (4.4)	8.5	26.7 (4.6)	11.3	25.8 (4.8)
* Highest attained education level*								
Bachelor or greater	27.5	25.9 (3.6)	33.4	26.4 (3.9)	26.9	24.7 (5.3)	36.0	24.4 (4.7)
Diploma	11.5	26.5 (4.4)	11.3	26.8 (4.9)	9.7	24.9 (5.0)	11.7	25.7 (5.6)
Certificate (trade/business)	26.7	26.9 (4.3)	25.3	27.7 (4.8)	13.1	25.6 (5.1)	14.5	26.3 (5.2)
School—Year 12 and below	34.3	27.3 (5.0)	30.0	27.8 (5.6)	50.4	26.4 (5.5)	37.9	26.8 (6.2)
* Occupation*								
Manager or professional	29.1	26.5 (3.9)	28.1	26.7 (4.2)	23.1	24.9 (4.9)	23.3	24.8 (4.8)
White collar	11.3	26.5 (4.7)	11.3	27.1 (4.6)	24.2	25.4 (5.8)	22.2	25.4 (4.9)
Blue collar	25.4	27.1 (4.8)	22.9	27.9 (5.1)	6.9	25.4 (5.4)	6.7	25.2 (5.4)
Unemp./not in labour force	34.2	26.7 (4.5)	37.8	27.2 (5.1)	45.8	26.3 (5.3)	47.9	26.5 (6.1)
* Household income (per annum)*								
≥ $130,000	7.2	25.8 (3.6)	24.7	27.0 (4.7)	6.4	23.9 (4.8)	22.7	24.5 (4.7)
$72,800–$129,999	27.4	26.9 (4.3)	34.4	27.3 (5.1)	25.9	25.7 (5.8)	30.3	25.6 (5.2)
$52,000–$72,799	22.6	27.1 (5.0)	13.5	26.9 (4.3)	19.8	25.2 (4.8)	14.5	26.0 (5.9)
$26,000–$51,599	27.2	26.4 (4.3)	20.3	27.6 (4.7)	27.4	25.7 (5.5)	21.8	26.6 (6.1)
$0–$25,999	15.7	26.8 (4.4)	7.1	26.9 (5.3)	20.6	26.7 (5.3)	10.7	26.7 (6.2)

Abbreviations: BMI, Body Mass Index; disadv, disadvantage; unemp, unemployed

^a^For each region, the countries of birth with highest proportion of respondents in 2006 were: **Oceania:** New Zealand, Fiji, Papua New Guinea; **North-West Europe:** United Kingdom, Netherlands, Germany; **Southern & Eastern Europe:** Italy, Poland, Fed Rep of Yugoslavia; **North Africa & Middle East:** Egypt, Lebanon, Turkey; **South-East Asia:** Philippines, Vietnam, Malaysia; **North-East Asia:** China, Hong Kong, Japan; **Southern & Central Asia:** India, Sri Lanka, Bangladesh; **Americas:** USA, Canada, Chile; **Sub-Saharan Africa:** South Africa, Mauritius, Zimbabwe.

### Neighbourhood disadvantage and mean BMI

#### Men

**Fig 1:**
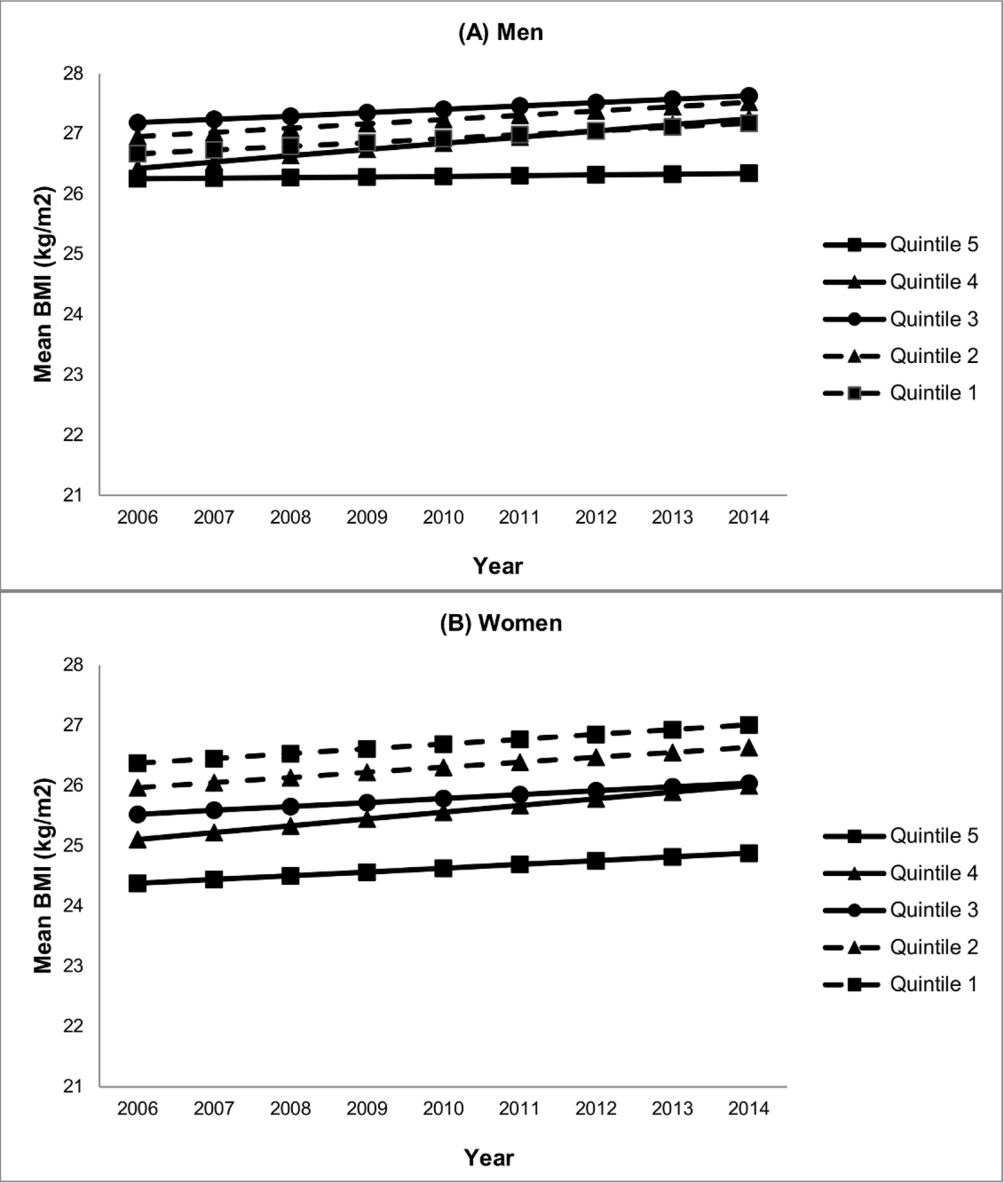
Immigrant BMI trends over time by quintile of neighbourhood disadvantage (2006–2014). (A) Men, (B) Women. Neighbourhoods in Quintile 1 are the most disadvantaged.

Table 2 shows the results of the step-wise regression modelling. After adjustment for age (model 1), mean BMI was significantly higher in men living in more disadvantaged neighbourhoods (in Quintiles 1, 2 and 3) compared with men living in the least disadvantaged neighbourhood (Quintile 5). These associations remained largely unchanged following adjustment for ethnicity (model 2), and individual socioeconomic position and geographic remoteness (model 3). In models 2 and 3, mean BMI also was significantly higher in Quintile 4 neighbourhoods than in Quintile 5 neighbourhoods (β = 0.55, 95%CI 0.07, 1.03). Fig 1A demonstrates the mean BMI trends over time by quintile of neighbourhood disadvantage. It illustrates the more rapid increase in mean BMI observed for men in Quintile 4 neighbourhoods compared with men in Quintile 5 neighbourhoods (β = 0.09, 95%CI 0.03, 0.16). The figure also shows a widening of BMI neighbourhood inequalities, arising from increasing mean BMI for all groups with the exception of male immigrants living in Quintile 5 (the least disadvantaged) neighbourhoods, for whom mean BMI remained almost static over time (0.1 kg/m^2^ increase from 2006 to 2014).

**Table 2:** Neighbourhood disadvantage and BMI for immigrant men and women, 2006–2014.

	Model 1^a^		Model 2^b^		Model 3^c^		Model 4^d^	
	Coeff	95% CI	Coeff	95% CI	Coeff	95% CI	Coeff	95% CI
***Men (n = 2*,*043 9*,*192 observations)***								* *
***Fixed effects***								* *
Intercept (se)	26.4	(0.189)	26.2	(0.215)	26.2	(0.242)	26.4	(0.253)
Time (0 = 2006)	0.057	(0.04,0.08)	0.058	(0.04,0.08)	0.059	(0.04,0.08)	0.012	(-0.03,0.06)
*Neighbourhood disadvantage*								
Quintile 5 (least disadvantage)	*Reference*		*Reference*		*Reference*		*Reference*	
Quintile 4	0.48	(-0.01,0.97)	**0.55**	(0.07,1.03)	**0.55**	(0.07,1.03)	0.18	(-0.36,0.73)
Quintile 3	**1.08**	(0.57,1.59)	**1.10**	(0.59,1.60)	**1.10**	(0.59,1.62)	**0.94**	(0.36,1.53)
Quintile 2	**0.98**	(0.45,1.49)	**0.94**	(0.43,1.45)	**0.94**	(0.41,1.47)	**0.70**	(0.11,1.30)
Quintile 1 (most disadvantage)	**0.65**	(0.13,1.17)	**0.63**	(0.12,1.14)	**0.62**	(0.08,1.16)	0.42	(-0.18,1.02)
*Interaction*								
Quintile 5*time							*Reference*	
Quintile 4*time							**0.09**	(0.03,0.16)
Quintile 3*time							0.04	(-0.02,0.11)
Quintile 2*time							0.06	(-0.01,0.13)
Quintile 1*time							0.05	(-0.02,0.12)
***Women (n = 2*,*250 10*,*212 observations)***	* *	* *	* *	* *	* *	* *	* *	* *
***Fixed effects***								* *
Intercept	24.2	(0.227)	24.7	(0.258)	24.4	(0.284)	24.5	(0.297)
Time (0 = 2006)	0.076	(0.05,0.10)	0.077	(0.05,0.10)	0.079	(0.05,0.10)	0.062	(0.01,0.11)
*Neighbourhood disadvantage*								
Quintile 5 (least disadvantage)	*Reference*		*Reference*		*Reference*		*Reference*	
Quintile 4	**0.94**	(0.35,1.53)	**0.89**	(0.32,1.46)	**0.92**	(0.35,1.79)	**0.72**	(0.08,1.36)
Quintile 3	**1.29**	(0.69,1.89)	**1.17**	(0.59,1.75)	**1.16**	(0.57,1.75)	**1.15**	(0.49,1.81)
Quintile 2	**1.93**	(1.31,2.55)	**1.71**	(1.11,2.31)	**1.67**	(1.05,2.29)	**1.59**	(0.89,2.27)
Quintile 1 (most disadvantage)	**2.31**	(1.71,2.91)	**2.14**	(1.55,2.72)	**2.08**	(1.48,2.68)	**2.01**	(1.34,2.69)
*Interaction*								
Quintile 5*time							*Reference*	
Quintile 4*time							0.05	(-0.02,0.12)
Quintile 3*time							0.00	(-0.07,0.08)
Quintile 2*time							0.02	(-0.05,0.10)
Quintile 1*time							0.01	(-0.06,0.09)

Abbreviations: Coeff, coefficient.

^a^Model 1: Neighbourhood disadvantage adjusted for baseline age, age squared and survey year.

^b^Model 2: Model 1 plus adjustment for ethnicity.

^c^Model 3: Model 2 plus adjustment for geographic remoteness, education, occupation, household income.

^d^Model 4: Model 3 plus interaction (neighbourhood disadvantage*year). Bold p<0.05.

#### Women

Table 2 demonstrates that among women, mean BMI increased significantly with increasing level of neighbourhood disadvantage. Adjustment for ethnicity (model 2) attenuated the differences somewhat, as did adjustment for individual socioeconomic position and geographic remoteness (model 3) for Quintiles 1, 2 and 3, although relationships remained significant. Fig 1B shows that over the period 2006–2014, mean BMI increased at a similar rate for all groups, effectively maintaining neighbourhood socioeconomic inequalities over time. Although there is some suggestion of a slightly faster mean BMI increase in women living in Quintile 4 neighbourhoods compared with women living in other neighbourhoods, this faster increase was not significant (β = 0.05, 95%CI -0.02, 0.12).

### Geographic remoteness and mean BMI

#### Men

**Fig 2:**
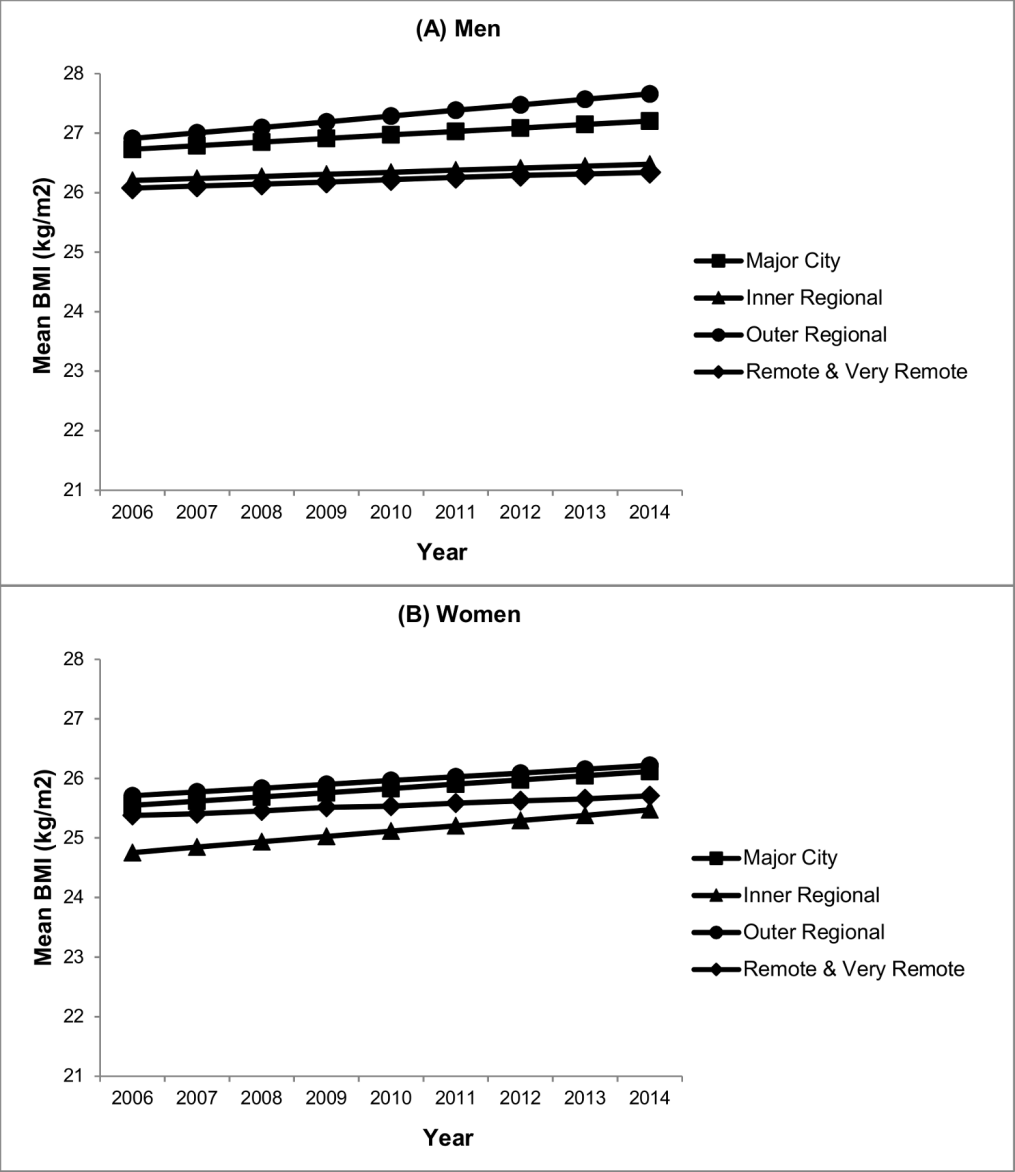
Immigrant BMI trends over time by geographic remoteness (2006–2014). (A) Men, (B) Women.

Table 3 shows the association between mean BMI and geographic remoteness. In model 1, men living in outer regional Australia had significantly higher mean BMI compared with those living in major cities (β = 0.79, 95%CI 0.08, 1.50). This difference was attenuated, however, and became non-significant following adjustment for ethnicity (model 2) and further attenuated following adjustment for individual socioeconomic position and neighbourhood disadvantage (model 3). As shown in Fig 2A, BMI increased at a similar rate over the period 2006 to 2014 for men living in all locations irrespective of the level of geographic remoteness.

**Table 3:** Geographic remoteness and BMI for immigrant men and women, 2006–2014.

	Model 1^a^		Model 2^b^		Model 3^c^		Model 4^d^	
	Coeff	95% CI	Coeff	95% CI	Coeff	95% CI	Coeff	95% CI
***Men (n = 2 043*, *9 192 observations)***								* *
***Fixed effects***								* *
Intercept (se)	27.0	(0.127)	26.8	(0.177)	26.2	(0.242)	26.2	(0.243)
Time (0 = 2006)	0.057	(0.03,0.08)	0.058	(0.04,0.08)	0.059	(0.04,0.08)	0.061	(0.03,0.09)
*Remoteness*								
Major city	*Reference*		*Reference*		*Reference*		*Reference*	
Inner regional Australia	-0.25	(-0.77,0.27)	-0.37	(-0.89,0.15)	-0.61	(-1.15,-0.08)	-0.52	(-1.10,0.06)
Outer regional Australia	**0.79**	(0.08,1.50)	0.60	(-0.10,1.30)	0.31	(-0.40,1.02)	0.20	(-0.59,0.98)
Remote and very remote	-0.25	(-2.02,1.52)	-0.65	(-2.38,1.09)	-0.74	(-2.46,0.99)	-0.62	(-2.60,1.37)
*Interaction*								
Major city*time							*Reference*	
Inner regional Australia*time							-0.03	(-0.09,0.04)
Outer regional Australia*time							0.03	(-0.07,0.13)
Remote and very remote*time							-0.03	(-0.27,0.21)
***Women (n = 2 250*, *10 212 observations)**** *								
***Fixed effects***								* *
Intercept	25.3	(0.150)	25.7	(0.208)	24.4	(0.284)	24.2	(0.306)
Time (0 = 2006)	0.077	(0.05,0.10)	0.079	(0.06,0.10)	0.079	(0.06,0.10)	0.071	(0.04,0.10)
*Remoteness*								
Major city	*Reference*		*Reference*		*Reference*		*Reference*	
Inner regional Australia	-0.10	(-0.75,0.56)	-0.26	(-0.90,0.38)	**-0.70**	(-1.34,-0.06)	**-0.80**	(-1.70,-0.12)
Outer regional Australia	**1.01**	(0.21,1.80)	0.71	(-0.07,1.48)	0.17	(-0.61,0.94)	0.17	(-0.69,1.03)
Remote and very remote	-0.08	(-2.03,1.88)	-0.20	(-2.08,1.69)	-0.32	(-2.16,1.52)	-0.14	(-2.18,1.89)
*Interaction*								
Major city*time							*Reference*	
Inner regional Australia*time							0.02	(-0.05,0.09)
Outer regional Australia*time							-0.01	(-0.11,0.10)
Remote and very remote*time							-0.03	(-0.27,0.21)

Abbreviations: Coeff, coefficient.

^a^Model 1: Geographic remoteness adjusted for baseline age, age squared and survey year.

^b^Model 2: Model 1 plus adjustment for ethnicity.

^c^Model 3: Model 2 plus adjustment for neighbourhood disadvantage, education, occupation, household income.

^d^Model 4: Model 3 plus interaction (geographic remoteness*year). Bold p<0.05

#### Women

In model 1, mean BMI was significantly higher in women living in outer regional Australia compared with those living in major cities (β = 1.01, 95%CI 0.21, 1.80). This difference was attenuated and non-significant following adjustment for ethnicity (model 2) and further attenuated after adjustment for individual socioeconomic position and neighbourhood disadvantage (model 3). In model 3, mean BMI was significantly lower in women living in inner regional Australia compared with those living in major cities (β = -0.70, 95%CI -1.34, -0.06). Fig 2B illustrates that over the period 2006 to 2014, mean BMI increased at a similar rate for all groups with no differences based on geographic remoteness.

### Discussion

This study reports new findings on BMI trends among an immigrant sub-group of Australian adults and their relationship with two elements of ‘place’ disadvantage: neighbourhood socioeconomic disadvantage and geographic remoteness.

### Neighbourhood socioeconomic disadvantage

Over the period 2006 to 2014, neighbourhood socioeconomic disadvantage was associated with mean BMI in both immigrant men and women, with a particularly strong relationship for women. The associations were robust to controlling for individual socioeconomic position and ethnicity, suggesting that neighbourhood socioeconomic disadvantage exerts an independent contextual effect on immigrant BMI. These findings build on research that found similar associations with the general Australian population [34–37].

To date, the literature has not considered BMI trajectories by level of neighbourhood disadvantage in an Australian immigrant cohort. Findings from this study are unique in demonstrating widening neighbourhood inequalities in BMI among men, arising from an increase in mean BMI for all groups over time, with the exception of immigrant men living in the least disadvantaged neighbourhoods. Among women, mean BMI increased at a similar rate for immigrant women across all neighbourhoods, and therefore, BMI inequalities between women living in the most versus the least disadvantaged neighbourhoods were maintained over time. These patterns of persistent or widening inequalities suggest that current obesity prevention interventions are not successful (or not yet successful) in reaching immigrants living in disadvantaged neighbourhoods in Australia. Comparable longitudinal research with the general Australian population is limited to two known studies. One study of a mid-older aged cohort living in Brisbane (the third largest city in Australia) found that, in both men and women, living in more disadvantaged neighbourhoods was associated with higher BMI and neighbourhood inequalities were maintained over time with all groups increasing in BMI at a similar rate [36]. The other study took a life course approach examining neighbourhood inequalities in BMI across different age groups in adulthood and found that neighbourhood inequalities were evident from 15–24 years and were maintained across age groups for men and widened for women [37]. Together these findings underscore the importance of further research with both the general population and immigrant cohorts to understand the (potentially different) underlying causes of neighbourhood inequalities in BMI [36,37]. Longitudinal studies of BMI and neighbourhood disadvantage among ethnic minorities in the US [16,28,29,31,62] are not directly comparable due to the differing immigrant cohorts, immigration histories, geographic settlement patterns and policy contexts between the two countries. Studies from other contexts can, however, suggest promising directions for future research on mediating factors that have been shown to be significant (and protective) in the relationship between neighbourhood disadvantage and trends in BMI in ethnic minority groups. These include aspects of the built environment, such as neighbourhood walkability [16]; elements of the socio-cultural environment, such as own-group neighbourhood ethnic density and social networks [28]; and life course considerations, such as addressing exposure to neighbourhood disadvantage during critical life-course periods [29,62].

### Geographic remoteness

This is the first known study of the relationship between geographic remoteness and BMI trends among immigrants to Australia. Over the study period, male and female immigrants residing in outer regional areas had significantly higher mean BMI compared with their counterparts in major cities. These differences were largely attenuated and no longer statistically significant following adjustment for ethnicity, individual socioeconomic position and neighbourhood disadvantage, suggesting no independent effect of living in outer regional areas on mean BMI. The implications of these findings are that policy interventions need to target high BMI among immigrants living in outer regional areas. However, it is not the ‘remoteness’ per se which should be the focus (for example improving access to services), but rather, obesity prevention interventions should consider the role of ethnic factors, individual socioeconomic factors and neighbourhood disadvantage and seek to intervene on these fronts.

Trajectories of change showed all groups increasing in BMI at a similar rate. Comparing findings of this study with trends in the general Australian population is problematic given that most other studies have been cross-sectional [39,40]. The only known cohort study used a life course approach and found higher BMI with higher accumulated exposure to rural residence, as well as identifying a potential sensitive period of exposure to rurality at ages 26–30 years being associated with obesity later in adulthood [41]. There are no comparable international studies with immigrants or ethnic minority cohorts, although obesity differences in rural vs urban areas in the general population have been identified as important in the US [33] and Finland [63].

Further research to build on the findings of this study is needed. In particular, future prospective studies could focus on the role of sociocultural environmental factors in explaining BMI differences by geographical remoteness. Although not yet tested empirically in Australia, it could be hypothesised that ethnic density or living among people of a similar ethnic group may strengthen social ties and provide some protection against racism (including inter-personal and structural racism [64]), which has been linked to obesity [65]. Further, maintenance of health-protective cultural traditions post-arrival may be easier in more densely populated metropolitan areas [66], where there are greater social supports and infrastructure (e.g. access to ethnic food stores, places of worship). Mixed methods or qualitative approaches with ethnic communities in cities and regional locations would be of benefit to explore these facilitators further.

### Strengths and limitations

This study has a number of strengths that advance the field of environmental influences on immigrant bodyweight. These include the longitudinal design to study two contextual factors and trends in BMI. Further, the analysis used a nation-wide sample of immigrants and recent survey data (2006–2014) that reflects contemporary, policy-relevant BMI trends.

There are also limitations to consider in interpreting the findings. The HILDA data has an under-representation of immigrants born in countries where English is not the main language (14.7 per cent of the original sample compared with population benchmark of 17.5 per cent) [67]. This group was also more likely to be lost to follow up and were more likely to be excluded from the analytic sample (primarily due to non-return of the self-completed questionnaire, rather than refusal to provide height and/or weight data). The direction of bias on regression estimates is unclear, depending on their association with BMI. For example, immigrants from North Africa/Middle East countries have been shown to have higher mean BMI and immigrants from South East Asian countries have been shown to have lower BMI [44]. Future studies with non-English speaking ethnic groups (using linguistically inclusive methods such as translated surveys, culturally-trained interviewers and bilingual interviewers[68]), would assist in assessing the extent to which the findings presented in our study can be generalised to this cohort. Self-reported BMI is subject to reporting errors and the magnitude of these errors may vary by ethnic group [69]. Also, the spatial scale used here to define neighbourhoods may not be the spatial area relevant for individuals in terms of contextual associations with BMI or BMI change [13], although recent Australian studies have used similar measures of the neighbourhood environment to predict engagement in health behaviours, like physical activity, that are protective against overweight and obesity [70]. Immigrant length of residence has been shown to be associated with BMI trends [16]; however, sub-analyses by length of residence in Australia was outside the scope of this study (and would be limited by sample size constraints). Finally, this study relied on census-derived measures of neighbourhood disadvantage and geographic remoteness to characterise immigrant neighbourhoods. While this is useful in describing overall patterns of area-level inequalities, more specific measures related to different dimensions of the built environment and socio-cultural environment would be of benefit in future research.

## Conclusions

This study is the first to demonstrate the existence and persistence of inequalities in BMI for immigrants living in disadvantaged neighbourhoods and in outer regional areas of Australia. The findings highlight the importance of multi-level obesity policy approaches that consider the environments where immigrants live, as well as the importance of designing interventions to be inclusive of immigrants living outside of capital cities. Further prospective research on area-level mediators is needed.
